# Midwife-centred management: a qualitative study of midwifery group practice management and leadership in Australia

**DOI:** 10.1186/s12913-022-08532-y

**Published:** 2022-09-26

**Authors:** Leonie Hewitt, Ann Dadich, Donna L. Hartz, Hannah G. Dahlen

**Affiliations:** 1 School of Nursing and Midwifery, Western Sydney University, Locked Bag 1797, Penrith, NSW 2751 Australia; 2 School of Business, Western Sydney University, Locked Bag 1797, Penrith, NSW 2751 Australia; 3 School of Nursing and Midwifery, University of Newcastle, Gosford Hospital, Level 9, 77a Holden St, Gosford, NSW 2250 Australia

**Keywords:** Midwifery-led care, Continuity of midwifery care, Management, Leadership, Sustainability

## Abstract

**Background:**

Midwifery group practice (MGP) has consistently demonstrated optimal health and wellbeing outcomes for childbearing women and their babies. In this model, women can form a relationship with a known midwife, improving both maternal and midwife satisfaction. Yet the model is not widely implemented and sustained, resulting in limited opportunities for women to access it. Little attention has been paid to how MGP is managed and led and how this impacts the sustainability of the model. This study clarifies what constitutes optimal management and leadership and how this influences sustainability.

**Methods:**

This qualitative study forms part of a larger mixed methods study investigating the management of MGP in Australia. The interview findings presented in this study are part of phase one, where the findings informed a national survey. Nine interviews and one focus group were conducted with 23 MGP managers, clinical midwife consultants, and operational/strategic managers who led MGPs. Transcripts of the audio-recordings were analysed using inductive, reflexive, thematic analysis.

**Results:**

Three themes were constructed, namely: *The manager, the person*, describing the ideal personal attributes of the MGP manager; *midwifing the midwives*, illustrating how the MGP manager supports, manages, and leads the group practice midwives; and *gaining acceptance*, explaining how the MGP manager can gain acceptance beyond group practice midwives. Participants described the need for MGP managers to display midwife-centred management. This requires the manager to have qualities that mirror what is generally accepted as requirements for good midwifery care namely: core beliefs in feminist values and woman-centred care; trust; inclusiveness; being an advocate; an ability to slow down or take time; an ability to form relationships; and exceptional communication skills. Since emotional labour is a large part of the role, it is also necessary for them to encourage and practice self-care.

**Conclusions:**

Managers need to practice in a way that is midwife-centred and mimics good midwifery care. To offset the emotional burden and improve sustainability, encouraging and promoting self-care practices might be of value.

## Background

Midwifery group practice (MGP) is considered the gold standard of care for childbearing women and their babies [1]. This model of care involves a group of midwives who support each other to care for a caseload of women throughout pregnancy, birth, and the early postnatal period [2]. MGP requires midwives to: be on-call; take responsibility for the ongoing care of women and babies; be autonomous; and work to their full scope of practice [3]. The alternative approach for employed midwives is generally shift-work, providing fragmented care for an aspect of a woman’s journey [4].

The majority of MGP models in Australia occur within the public hospital system, although they may be based in the community or in the hospital [5]. There are a range of models available, with a variety of ways the model is operationalised regarding: how the midwives work on-call; how many midwives work in each practice and which women may be offered the service including their level of complexity [6]. Varieties in MGP may be the result of local demographic requirements, local changes to suit the service or midwives and differences depending on how the model is interpreted. Within Australia there is no nationally agreed way of reimbursing the midwives who provide this care, with salaries differing within and between states and territories [5].

In MGP, women can form a relationship with a known midwife, improving both woman and midwife satisfaction [7–9]. Birth outcomes for women are also improved, with women experiencing fewer interventions during childbirth, higher spontaneous birth rates, fewer premature births and reduced foetal and neonatal loss [7]. MGP can positively influence midwives’ work satisfaction and reduce burnout, helping to retain them in the profession [8, 10–12]. There are also positive benefits for health services that offer MGP, including cost benefits [13], reduced MGP midwife sick leave, as well as the attraction and retention of staff [4]. Yet the model is not widely implemented and sustained, resulting in limited opportunities for women and midwives [14]. While MGP has grown over the past twenty years, only about 15% of births in Australia occur under caseload care [15].

Although researchers have considered the sustainability of the model, many have examined this through the lens of the midwife, maternity service, and woman by investigating burnout, cost, satisfaction, and birth outcomes [1, 4, 8, 9, 13, 16–19]. There are many factors that hinder implementation and sustainability; for example: funding; support; and insufficient midwifery staff availability [14]. However, one that has received limited scholarly attention is the impact of how these models are managed. Although, Dawson and colleagues [14] explored managers’ views on their intention to implement an MGP, little attention has been paid to how MGP management and leadership shapes the sustainability of the practice.

Despite the interrelatedness of management and leadership, the two concepts are different [20, 21]. Managerial roles require individuals to control and direct staff, resources, structures, and systems to achieve organisational goals [21]. Leadership is generally not a position, but an action that influences, motivates, and inspires others. Leadership is often associated with vision, integrity, commitment, risk-taking, and the ability to communicate [20]. Managers are employed to manage, and many might be expected to lead; however, not all managers are leaders [21]. Leadership in healthcare is generally viewed from a leader-centric perspective, where leadership is expected to be enacted from hierarchical positions. Rarely are the combined efforts of the collective seen as being responsible for leadership throughout the organisation. However, taking the attention away from the individual and placing the work of leadership on shared collective patterns of action can be more appropriate [22], especially within a group of passionate, highly qualified, professionals.

## Methods

The aim of this study is to clarify what constitutes optimal management to ultimately sustain MGP in Australia. As part of a larger mixed methods study, this is achieved by consulting with MGP managers, clinical midwife consultants (CMCs), as well as strategic, and operational managers.

An interpretive qualitative approach was employed. Specifically, a focus group was facilitated with CMCs who held leadership roles in MGP models; additionally, interviews were conducted with MGP managers, one CMC, as well as operational and strategic managers of services with MGP models. These interviews and focus group represent phase one of the larger study, which informed a national survey to investigate what constitutes optimal management of MGP in Australia.

Participants were recruited through social media and word-of-mouth. Those who expressed interest in participating were sent information on the study and a consent form to sign. They were also invited to complete a demographics survey via email. No participants dropped out of the study, and all were interviewed at their workplace, face-to-face or via web-conference, pending their preference. No interviews were repeated and transcripts were not offered to the participants for feedback, however, participants were asked to give feedback on the survey pilot derived from this study. Those eligible to participate were midwives who had held the following positions in the last five years to optimise the currency of the findings (see Table 1).

**Table 1:** Participants

**Position**	MGP manager	CMC	Operational and strategic manager
Participant Number	5	1 14	3
Method	1 face-to-face Interview 4 web-conferences	1 face-to-face Interview 1 face-to-face focus group	2 face-to-face interviews 1 web-conference
Duration with MGP responsibilities	Range: 2–8 yrs Average: 3.8 yrs	Range: 6 wks-17 yrs Average: 5.3 yrs	Range: 3–6 yrs Average: 5 yrs
Age	Range: 38–51 yrs Average: 48.2 yrs	Range: 33–63 yrs Average: 50.0 yrs	Range: 43–56 yrs Average: 51.0 yrs
State or territory	Victoria, Tasmania, New South Wales, Northern Territory	New South Wales	New South Wales
Place of birth	Australia: 4 United Kingdom: 1	Australia: 12 United Kingdom: 2 Central Europe: 1	Australia: 3

Each interview and the focus group took between 27 and 62 min. Only the researchers and participants were present. The schedule of questions encompassed what the participants saw were necessary characteristics for the MGP manager role, (see Table 2). The questions were built from previous studies looking at the role of the MGP manager [23, 24]. Information that could identify the individuals who participated was changed to protect their anonymity and pseudonyms were used. Ethics approval was granted through relevant human ethics committee (approval number: H13428).

**Table 2:** Participant questions

Position	Questions
MGP manager	• What do you do to support the work of MGP midwives?
	• What characteristics do you have that you think are important in being an effective manager?
	• What facilitates or restricts your role as a manager?
	• What factors influence the sustainability of an MGP?
CMC	• What have you observed helps support the work of MGP midwives?
	• How have you seen managers play a role in this?
	• What characteristics do you think are important in a manager of MGP?
	• What are the factors that influence sustainability of an MGP?
Strategic and operational manager	• What characteristics do you think are important in a manager of MGP?
	• Do you consider certain characteristics when employing an MGP manager that are different for other maternity unit managers and if so, what are these?
	• Why do you think it is important for this facility to have an MGP?
	• What are the factors that influence sustainability of an MGP?

## Data analysis

Data were analysed thematically, as described by Braun and Clarke [25], and assisted by the software program, Quirkos [26]. Inductive, reflexive thematic analysis (TA) was conducted to analyse data from the ground up, rather than using existing theory to mould the analysis [27]. Inductive analysis is often shaped by the researcher’s knowledge and experience, necessitating the use of reflexivity. According to Braun and Clarke, reflexive TA involves the researcher, who through growing engagement with the data, shapes the analysis via a flexible, organic, iterative process [25, 28]. The researcher is deeply involved in the reflexive, interpretation of the data, using the act of coding to interrogate the data and find nuanced or implicit meaning [29].

Familiarisation with the data occurred by reviewing the audio-recordings several times, while checking and re-reading the transcripts and researcher notes. Interviews were numbered and then later replaced with pseudonyms. Coding was a fluid, iterative, and recursive process during which themes were constructed that were shaped and reshaped using creativity and reflexive interpretation. The authors defined, reviewed, and honed the themes using thoughtful and analytic engagement with the data. Since three authors had extensive experience in MGP, the insider perspective was noteworthy and often moderated by the outsider perspective of the remaining author, who was not a midwife. The three insiders had held positions akin to the participants, many of whom were known to them through the close network of midwifery. The first and fourth authors conducted the interviews to help equalise the power dynamics between the interviewer and interviewee. These authors used their insider knowledge to respectfully pose probing questions and encourage explication. This blend of insider and outside lenses offered a balanced and complementary approach to the analysis.

Because TA is not conducive to conventional ways to determine sample size – like data saturation [29] – data collection ceased when information power was indicated. Information power suggests, ‘the more information the sample holds, relevant for the actual study, the lower amount of participants is needed’ – [30]. As such, data were analysed shortly after collection with reference to the study aim, sample specificity, use of established theory, quality of dialogue, and analysis strategy.

## Results

**Fig. 1:**
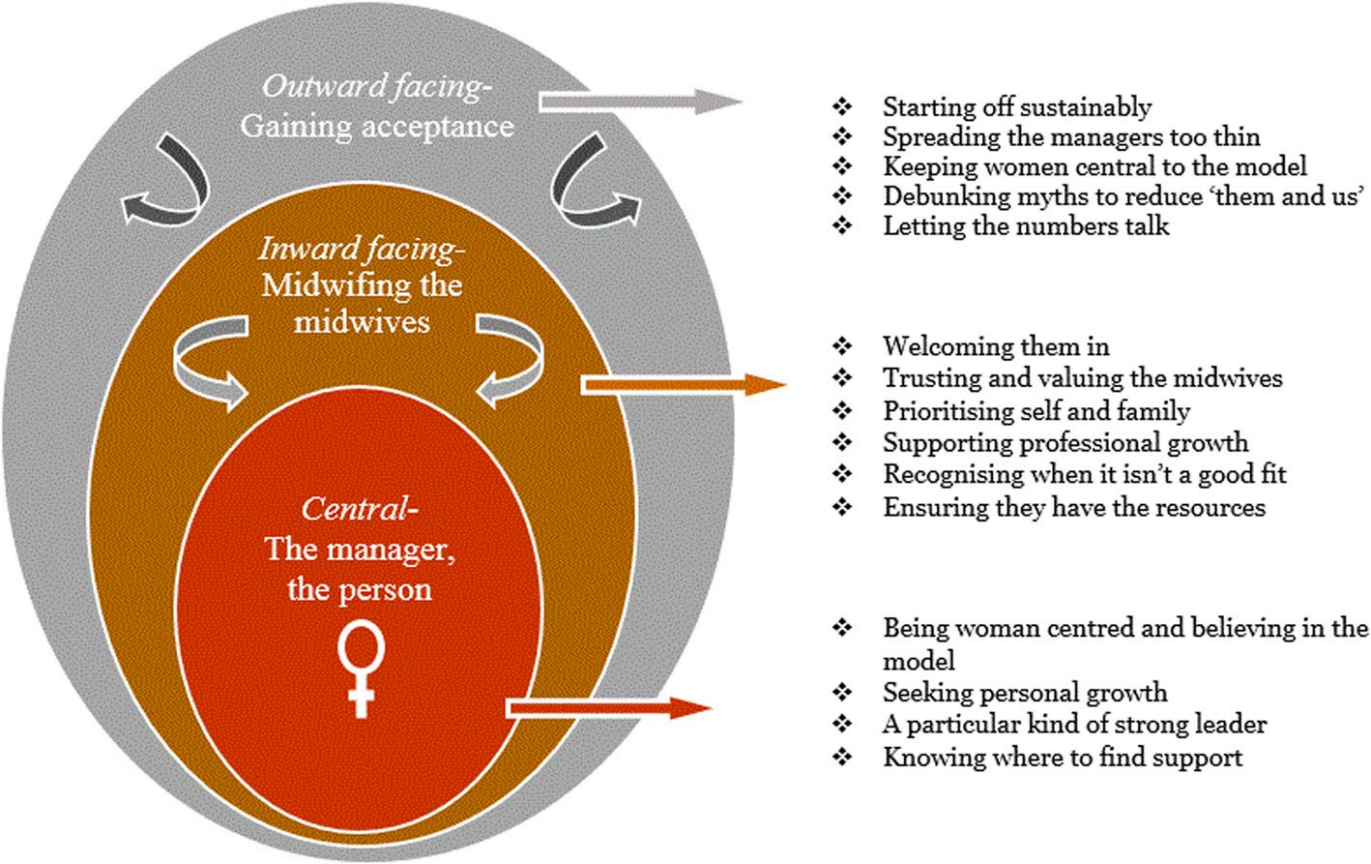
Thematic themes and subthemes

Following a thematic analysis of the data from interviews with MGP managers, executive managers, and CMCs, a central theme was constructed (see Fig. 1), namely, ‘the manager, the person’, describing the MGP manager’s ideal personal attributes. Moving out from the central theme is the inward facing theme – ‘midwifing the midwives’, illustrating how the MGP manager supports, manages, and leads the group practice midwives. The third and outward facing theme – ‘gaining acceptance’ – explaining how the MGP manager can gain acceptance by improving support outside the group practice.

### The manager, the person

#### Being woman centred and believing in the model

The central theme – the manager, the person – captured what participants described as an MGP manager’s ideal personal qualities. This theme encompasses woman-centredness and believing in the model, seeking personal growth, being a particular kind of strong leader, and knowing where to find support. These sub-themes are addressed in turn.

The participants indicated that managers who are woman-centred positively influence midwives’ care. Managers who held strong feminist, woman-centred values can influence others by role modelling and using their values to guide decisions. Furthermore, they were more likely to operationalise a service that was offered to all women regardless of risk:

I believe that every woman should have access to a known midwife… we should progress the model as an all risk… the women who probably need that known midwife maybe more, are those women who have challenges in their pregnancy (*Jenny, MGP manager*).

We are robbing so many women of that [MGP care] and that’s what makes you powerful as a woman. That’s what makes you a mother, that’s what makes you successful in your job… in the bedroom it changes you (*Dianne, strategic/operational manager*).

Finding managers who were woman-centred and who understood and believed in the benefits of the model for women and midwives was seldom easy. To be a good role model and optimally manage an MGP service, the manager needed these qualities. Christine described the questions she asked to find managers who had that belief, understanding, and philosophy:

I tailor my interview questions... I ask them what their understanding of midwifery group practice is and the benefits of it. My expectation that the answer… would be very much around the high rates of normal birth, the increased satisfaction for women, the increased breastfeeding rates. But also… that they have an understanding of the benefits for midwives *(Christine, strategic/operational manager).*

However, not all MGP managers started with a belief in the service. Some needed to witness the benefits to believe in the model. Hannah described how the MGP manager grew to believe in the model after starting as a non-believer:

The MGP MUM [manager] stepped into the model without the belief. She is the biggest advocate now, the biggest believer (*Hannah, strategic/operational manager*).

MGP was not always understood or supported. Many MGPs faced threats from those with power and little understanding of the benefits, or regard for what women wanted. Managers who understood the model, believed in the benefits, and had woman-centred values were more likely to fight for the model:

The manager has to be really strong [and have] belief in the model to fight for it (*Dianne, strategic/operational manager*).

#### Seeking professional growth

While formal education in management and leadership might be beneficial, it was not a prerequisite for MGP manager positions. Personal growth in areas the participants thought were limited provided MGP managers with confidence to optimally manage the MGP. Although many had not completed formal management and leadership education, they recognised its benefits for them, the model, and the profession:

Most of us in nursing and midwifery, we're not taught those leadership skills. I mean, I did do a diploma in management… I think there is a place for people to have an understanding of the different types of leadership that you can do within the workplace. It could be quite valuable to have leadership skills *(Michelle, MGP manager).*

Another area deemed as important for the MGP manager was financial management. Often, the reasons for not implementing or closing an MGP was its perceived expense. Financial management skills could enable managers to expose the financial benefits of an MGP service and manage the service in a financially judicious way:

Developing the skills of our midwifery unit managers [is important]…. none of us had any kind of cost centre or financial management training before we went into these roles (*Christine, strategic/operational manager*).

##### A particular kind of strong leader

The participants described components of what they classed as leadership, along with qualities that were nurturing. They described the need for the MGP manager to have qualities like strength and vision. However, being mother-like and humble were also important.

To have… confidence, you also have to have a certain amount of humility and you also have to have vision I think in being able to see where a model can go (*CMC, focus group*).

To be mother-like, but yet have the strength to address performance issues and clinical issues. To be nurturing because caseload’s a lifestyle, it’s not just a job (*Dianne, strategic/operational manager*).

The participants spoke of the importance of: having a vision and the ability to share that vision; inclusiveness; being inspirational; and having passion. For example, they needed to be strong while remaining gentle, set the cultural tone, and inspire the team with a shared direction. Managers who led by including group practice midwives in service planning also encouraged ownership:

Your main mission is to role model, to get people to see your vision, and to encourage them to come with you, and see that that's the way that we want to work... They're approachable, they're open, they're enthused, they're encouraging *(Michelle, MGP manager).*

An inclusive leadership perspective where, if everybody’s got the shared goal, people have ownership of what we’re trying to achieve; they feel a part of it (*Katie, MGP manager*).

Strength of character came in different forms. Susan described the necessity to be flexible, have good communication skills, to be honest, and to be fair. However, being accessible and welcoming were necessary components of this strong manager:

I'm very accommodating, I'm very approachable. I'm fair, I'm honest. I see that having good communication skills and being available is really important to manage the MGP service (*Susan, MGP manager*).

Being flexible in how midwives worked and who they worked with were also important considerations for the manager. Choosing who they partnered with allowed them to work with colleagues they trusted and who shared similar philosophies. Participants described how it was important for the manager to be flexible around the way midwives’ wanted to work, including how they operationalised on-call work:

Some people want to be on-call, want to be at their woman’s birth… but some people are like, ‘No, if you’re off, you’re off’ (*Anne, MGP manager*).

Sustainability is flexibility around how the midwives want to work. So, some do 24 hours on-call, some do 12 hours on-call, some do seven days of nights, others do night by night (*Christine, strategic/operational manager*).

##### Knowing where to find support

Support from executive management was critical to the wellbeing of the MGP manager. Since the MGP manager role differed from that of other managers within a facility, they sometimes experienced isolation. Furthermore, MGP managers were required to have a nurturing, caring, and compassionate personality, which might mean they are more vulnerable and potentially required added support:

We have to protect her because with that… nurturing personality comes quite an emotional person as well. She’s not a hard nut so, much as she is there for the caseload midwives, when the tears come, her [operational manager]… and I [the strategic manager] try to be there for her (*Dianne, strategic/operational manager*).

Not all managers had supportive executive management. Some participants found support from the midwives they managed or from other staff who were friends. Some had access to people who had previously held the manager position, while other MGP managers found their partners an important source of support:

I did tap into people who had previously managed a group practice or who’d had previous experience with a group practice… it was a mentorship in an informal way (*Jenny, MGP manager*).

He doesn’t understand, but… having good support systems at home is really important (*Anne, MGP manager*).

### Midwifing the midwives

This inward facing (looking at the group practice) theme encompasses participant views on how the MGP manager midwifed the midwives. Midwifing the midwives refers to the careful, thoughtful, and compassionate management of the group practice midwives. Coined by Brodie [31], midwifing the midwives describes caring for midwives by mirroring how midwives work with women. This approach does not assume to know what is best for them or tell them what to do; however, it can involve shielding them while carefully listening, so they might find their own solutions. The participants explained how MGP managers should midwife the midwives, through the following themes:

#### Welcoming them in

Participants explained that communication was a large part of the MGP manager role. Having a style of communication that was open, honest, and transparent can build trust and relationships. Having an open, non-judgemental, and honest style, while being kind and approachable were essential. Midwifing the midwives typically required the manager to be available and approachable to MGP midwives:

The manager needs to be able to have those communication skills that enable a conversation to start… and not making her feel judged, or anything like that, making her feel safe to be able to express whatever it is that she’s concerned about (*Hannah, strategic/operational manager*).

If they need you or if something has happened in the birth suite, then I need to be available to have a debrief (*Susan, MGP manager*).

Relative to other health service managers, MGP managers were required to be perhaps more vigilant to detect signs of stress from being on-call, as well as the responsibility and autonomy associated with the MGP midwife’s role. The participants described the need for the manager to be welcoming and nurturing. For instance, Alex described the need for the manager to be looking for signs of stress that might not be immediately obvious, while Michelle discussed the need for midwives to feel safe to discuss anything:

The manager needs to be like a port master who welcomes in the ship… never knowing what they bring… They may bring fantastic news and joyful reminiscence, or they may bring stories of tragedy… [the manager] should be the stoic and steady on land beacon that welcomes them. Somebody who is observant and can see when they are struggling (*Alex, CMC*).

I think that's the number one thing and be really open, have a great open-door policy that people can approach you and not be afraid to discuss stuff (*Michelle, MGP manager*).

#### Trusting and valuing the midwives

Participants described how midwives needed to feel valued to continue to provide care in a model that required a considerable commitment. They suggested that managers should understand the commitment midwives made to work in the MGP and the sacrifices they made on their family and social life. Ensuring the group practice midwives felt appreciated and valued might help to retain them:

[MGP managers] need to understand the demand of being on-call. Even if they didn’t experience it, they need to have a strong appreciation for the on-call and for the effort it takes to be on-call… They have to see midwives that come into those roles as special and advocate for them as such (*Alex, CMC*).

You want them to stay in caseload and I know that a big part of them not staying is because they feel that they’re not appreciated (*Anne, MGP manager*).

Trusting midwives and managing them with a hands-off approach were essential to grow a healthy culture within the MGP group. Participants indicated that managers can show they trust the midwives by not micro-managing them and appreciating the need for them to be autonomous. They also described how autonomy can attract midwives to MGP:

They’ve got to trust that the midwives can sort out their workload, sort out how they’re going to work, when they’re going to see the women, when they are going to have downtime, all that. They’ve got to trust in that, and if they don’t, it’s not going to work if they try to micromanage everything (*CMC, focus group*).

Part of the attraction, why they want to work in the model, is because it gives them autonomy (*Anne, MGP manager*).

Because the MGP midwives relied on each other for support, it was important that they regularly met to bond. Valuing the midwives involved prioritising regular meetings. MGP meetings also provided an opportunity for: case review with medical staff; connecting with the midwives; education/updates; and relationship building:

We would have regular meetings... bringing the group back together, so we could all consolidate and reconnect. It was about developing the culture within the team that we were there for each other if needed, and we had each other's back (*Katie, MGP manager*).

#### Prioritising self and family

In a service where midwives might be at increased risk of experiencing stress, the participants indicated that the manager must prioritise the midwives’ self-care to sustain the model. Encouraging self-care and implementing strategies to help midwives recognise and prioritise personal needs can sustain and retain staff. Midwives needed to care for themselves first, so they can care for the women:

They had to come to me within their monthly catch up and tell me what they were doing in line with self-care… getting them to think about looking after themselves so that they could look after the women in their care as well (*Jenny, MGP manager*).

There’s actually a need for formal supervision. A very good friend of mine was a manager of [a mental health service. She explained that]… when she was having a tough time about various bits and pieces… it’s a natural thing for them to go and have supervision sessions (*Katie, MGP manager*).

The participants also explained that the MGP manager must recognise the need for midwives to prioritise their families. Midwives were unable to properly care for women if preoccupied with home-related concerns. It was important for managers to recognise that midwives might have complex and dynamic caring responsibilities at times and that midwives must put their families first:

To even consider working a caseload or working on-call, you really need… the support from home. You need to have the ability to be flexible… You don’t want to be justifying why you’re going to drop everything and walk away and go and work (*Alex, CMC*).

#### Supporting professional growth

Encouraging professional development of the MGP midwives was part of the MGP manager role. This included taking an active interest in their professional growth, ensuring they fulfilled their requirements as an MGP midwife, and encouraging the professional development of newer group members. This served to keep midwives stimulated and drive evidence-based change:

Keeping midwives engaged and motivated, focusing on empowering the midwives to build skills and capacity… That could be in auditing, that could be in working. ‘Okay, you’re passionate… about optimal cord clamping. That clinical guidelines are up for review. Why don’t you have a look at it?’ (*Katie, MGP manager*).

According to the participants, supporting early career midwives and midwives new to MGP was an important part of the MGP managers’ role. Newly graduated midwives and midwives who had provided fragmented care from core services can find the transition to an MGP model challenging. Because recruitment to the service was necessary for sustainability, and support for these midwives was essential to them staying, Christine explained that managers needed to provide bespoke support:

I can think of… one midwife… who has been a midwife for over 20 years. Very experienced birth unit midwife but hasn’t done antenatal care for probably 15 years... it’s been really, really challenging for her …The other thing about sustainability of MGP is supporting our less experienced midwives into that model (*Christine, strategic/operational manager*).

#### Recognising when it isn’t a good fit

Sometimes the model did not work for individual midwives. While there were many reasons that MGP might not work for an individual, it was often due to the on-call requirements, family commitments, social commitments, increased autonomy or responsibility, or poor cultural alignment between an individual midwife and the group practice. The participants described that when midwives were not a good fit for MGP, it was often recognisable within a short time:

There are some midwives… not really suited to the model because their needs are greater than what the model can provide… they don’t tend to last very long in the model… So, we’ve had midwives come on to the model and last 12 months, six months, hasn’t worked out. We can sniff out the issues pretty quickly (*Hannah, strategic/operational manager*).

Addressing the poor cultural alignment between an individual midwife and the group practice was an important part of MGP management. Although it was never easy for managers to remove midwives from the model, it was sometimes necessary to sustain the MGP:

It has to do with the dynamics of the groups… it could have fallen down because it’s just not working with those people, or you’ve just got one person that isn’t really fitting into the service. It’s too much for them or they just find it too emotionally hard, and it’s recognising that and being able to deal with it. And it might mean moving somebody into another group or taking somebody actually out of the MGP (*CMC focus group*).

#### Ensuring they have the resources

A successful MGP required resources, including equipment, tools, space, rooms, and staff. Ensuring midwives had tools to work helped them to use their time more efficiently and be with the woman. Keeping an MGP well-resourced with equipment and facilities also involved making the most of opportunities as they arise within the hospital or community. An example of this was repurposing rooms in the community, like early childhood centres, and making use of charitable organisations that supplied equipment:

I think it’s really helpful to have a good understanding of how to utilise resources within health systems… [the MGP managers] are the key person to making these models sustainable and successful (*Christine, strategic/operational manager*).

It’s really important to have our own space. When I worked in [another hospital]… we didn’t have our own space… Here we have a building and… consulting rooms that we use and now the obstetrician works from there on Monday for any women who need to be referred. It just happened to be there at that time and were able to negotiate it (*Michelle, MGP manager*).

A key resource a manager needed to prioritise was staff. Keeping the MGP well-staffed ensured MGP sustainability. However, MGP is a service that might not suit midwives during some stages of their life, like pregnancy or when their children were young. It was therefore necessary to accept staffing changes within the model, rather than consider these are failures:

You’ve got to always be growing and you’ve got to be relaxed about the maternity leave and then the comings and goings (*Dianne, strategic/operational manager*).

### Gaining acceptance

The theme, gaining acceptance, described the outward facing (away from the group practice) strategies that improved sustainability. These involve starting the MGP in a way that promoted sustainability, but avoided spreading the managers too thin, while keeping the women central to the model. The manager was also responsible for debunking myths about MGP by educating stakeholders, reducing the siloed culture that fragments healthcare, and letting the numbers talk. These sub-themes are elucidated.

#### Starting off sustainably

Participants described the importance of establishing the service to be sustainable from the outset. This involved having a manager and enthusiastic midwives to implement the model. Alex described how important it was to have the support from a manager and Katie described the importance of having experienced midwives to support the model and the manager:

We really struggled with having that support at the beginning… When we had a manager that actually was able to [support us]… it made such a big difference (*Alex, CMC*).

[The MGP midwives] brought a wealth of experience with them… because everybody had done some homebirth at some point… They helped support my leadership and helped me grow to a certain extent as well. Every voice was equal in the team (*Katie, MGP manager*).

Being sustainable required a strategic plan. For some participants, this involved starting small and building on the practice, once trust was established. For instance, it was helpful to start with a small group of midwives or a low-risk model; then, while growing the model, adding services for example: homebirth; an alongside or a freestanding birth centre; or moving to an all-risk model.

We started out with a very small vision but always in our proposal we had room for expansion… and then we brought in home birthing. So, we didn’t put it in the one basket together right at the beginning because the obstetricians were very nervous about it all. But we gained their trust and that’s how we moved on (*Michelle, MGP manager*).

Many services became unsustainable when the main supporters moved on. A critical aspect to sustainability was ensuring the model was supported by the system, not by individuals. It was important to ensure that support was embedded in the way the model is operationalised:

Core people leave and models fall over, which is why it’s critically important that anything that we do with this model, the operations of this model has to be embedded in a way that is sustainable (*Hannah, strategic/operational manager*).

#### Spreading managers too thin

It was important to establish an MGP service in a way that afforded the manager the ability and time to manage it. Many MGP managers were given other areas to manage, partly because the MGP was seen as requiring little management. However, this meant the areas that require more managerial energy were prioritised to the detriment of the MGP. Consequently, MGP midwife retention and sustainability was put at risk along with the manager’s wellbeing:

I manage the MGP, antenatal, and the postnatal home care and also the other components of our service, so I’m very time poor for probably everything’ (*Susan, MGP manager*).

I came from a world, it was just the manager for the MGP, whereas my second world, I was the manager with an outpatient service and other things I discovered along the way once I started. So, it’s very hard if you’re not just the manager for that service. You have so many other focuses (*CMC, focus group*).

Some managers also managed areas that divided loyalties and attention – consider the manager who oversaw a birth unit as well as an MGP. The manager had to prioritise their time for the service that required more input and was viewed as essential; this raised dilemmas when issues occurred between the birth unit and MGP staff:

When you have a birth unit manager [who also manages MGP]… sometimes the priorities of birth unit takeover and we’re having a little bit of argy-bargy… between caseload and birthing unit (*CMC, focus group*).

It just doesn’t work because those managers are stretched, they have the finite amount of resources and they’re suddenly managing a model that goes across all of those areas (*Christine, strategic/operational manager*).

#### Keeping women central to the model

For the service to be woman-centred, participants explained the service needed consumer involvement. Having consumer representation was the only way a service could know if it met the needs of childbearing women. One way to help the service to focus on the woman was to encourage women to actively participate in forums where they can influence decisions, like working parties and steering groups. Participants described the importance of seeking and using the feedback from women:

You make time to… go and chat to the women. ‘Oh hi, I’m the manager from caseload… how was everything?’ (*Anne, MGP manager*).

Women need to drive the need for it as well and keep giving us the feedback, keep supporting us, keep providing some input for us as well, because they’ll drive it (*Hannah, strategic/operational manager*).

According to the participants the manager needed a woman-centred focus to ensure the service was geared around the woman’s needs. Because the manager was the interface between other services and the MGP, it was the manager’s responsibility to keep bringing the focus back to the woman instead of the system:

She’ll just maybe some say something, ‘What about the woman in that situation?’ and everyone says, ‘Oh yeah’ (*CMC, focus group*).

We really need to refocus the care on the needs of the women because no matter what we say, most large systems are geared towards maintaining the system, not the client or patient or woman… for caseload to become the main focus of the care, we really need to refocus our effort on the women (*Alex, CMC*).

#### Debunking the myths to reduce ‘them and us’

A large part of the manager’s role was educating and marketing MGP to stakeholders. Because the service was operationalised differently to traditional, fragmented, shift-based care, it was difficult for people to understand. Participants discussed how the manager needed to stop myths quickly that circulated within the hospital and educate others on what MGP offered:

I don’t really think we spend enough time educating the staff and smashing out the myths around what group practice is. ‘I don’t see them here ever’. They don’t have to come in and work eight hours like you do, but they might have done 60 hours last week (*Jenny, MGP manager*).

The essence of it all really is, keep marketing… keep it in people’s faces. ‘This is the way we function. This is our core business’ and ‘Isn’t this amazing’ (*Hannah, strategic/operational manager*).

MGP managers’ operational requirements were usually less than they were for other managers, giving them the capacity to communicate with other departments, stakeholders, and managers. Improved relationships between managers of maternity services offered opportunities to negotiate and avoid difficulties between core and MGP midwives, while being the visible face of the MGP service:

I see… [the manager] as the gatekeeper, ensuring that the other services and our other key stakeholders understand the work that… [MGP midwives are] doing and become that visibility for them (*Christine, strategic/operational manager*).

An example of an operational strategy that improved long-term relationships with core staff was to rotate them onto the MGP to relieve staff during extended leave. This enabled core staff to experience MGP before committing to the role and improved relationships and understanding:

If you get the core midwives to rotate in as a relief midwife… they actually get to see what it’s like, the positives and the challenges. They then take the experience back to the core service (*Katie, MGP manager*).

This strategy also reduced the perception that the woman was the sole responsibility of the MGP midwife, normalising the MGP within the institution. A CMC focus group participant described how MGP women were often treated as though the core hospital services had no responsibility for her care:

I’ve worked in MGP models where they’ll just put the woman in the room and the core staff don’t go anywhere near the MGP woman… The midwife’s coming in, she could have had a car accident, be two hours away, but they still leave her in that room (*CMC focus group*).

#### Letting the numbers talk

Promoting the MGP to executive managers by collecting and using statistics aided their education on the advantages of MGP. Statistics were also used to dispel rumours. Because statistics gave a numerical (and seemingly objective) reflection of what has happened, they can be a valuable tool for MGP managers:

Giving them all the feedback that we get and showing them data… Outcomes are very good… So, we’ll just keep pushing them up there (*Hannah, strategic/operational manager*).

Try to avert conflict by… things like statistics… because all that information is really transparent now (*Anne, MGP manager*).

It was also beneficial for midwives to be aware of their individual statistics, so they recognised areas they might improve and areas they can be proud of. Since the MGP role required the midwife to provide most of the care across the continuum, their individual perinatal statistics reflected their care more than most other practitioners who provided a fragment of the woman’s care:

Definitely capturing stats was part of that monthly meeting… so that we could make sure that we were meeting the KPIs [key performance indicators] of course but showing the positive outcomes with data (*Jenny, MGP manager*).

Another way to let the numbers talk was to show the financial benefits of MGP. Because MGP typically drew resources across several cost centres, the financial benefits were not always apparent to other managers – this was partly because MGP midwife wages were typically higher than core midwives. It was therefore important to show other stakeholders that MGP was cost-effective:

You’ve got to be brave, and you’ve got to be pretty savvy in your ability to demonstrate the benefits… Not just the benefits to women, but the budget and the money. People want to know about money. And so, you’ve really got to demonstrate how cost efficient this model can be (*Hannah, strategic/operational manager*).

## Discussion

Although MGP provides the ‘gold standard of care’ [7], little is known about how to manage, lead, and ultimately sustain it. Many MGP services are not implemented or sustained, diminishing women’s access to this service [15]. This study clarified what is required to optimally manage and lead an MGP to improve its sustainability.

In this study, 23 managers and CMCs of Australian MGPs participated in interviews or a focus group. The themes generated included: *the manager, the person; midwifing the midwives; and gaining acceptance.* These themes described midwife-centred management that mirrors what is frequently described as optimal midwifery care (woman-centred care). Participants described the need to be emotionally available, to be trusting, to slow down, to advocate, and to form meaningful relationships – all characteristics of good MGP care [32]. While midwives put women at the centre of care, the participants described how ideal MGP managers are required to put the midwives at the centre of their management. According to the participants, managers were able to manage the MGP in this way because they: believed in midwifery; trusted in midwifery care; and held a deep-seated belief in feminist values and woman-centred continuity of care. Although health service managers are required to meet organisational obligations, the managers in this study indicated their focus was on the midwives, putting them front and centre, enabling the midwives to provide woman-centred care, and thus sustaining these gold standard models.

### Mirroring good midwifery care

Relational midwifery is a consistent thread through MGP. Whether it is described metaphorically, as the glue that holds it all together [32], or the hidden warp threads of a tapestry [33], relationships are the foundation of MGP and good midwifery care. However, relationships also form the basis of effective management of MGP. A previous study described the ability to form relationships, as crucial to MGP management [34]. Forming strong interdisciplinary relationships with stakeholders, other departmental managers, hospital staff, and the MGP midwives is essential to the MGP managers’ role [24, 34]. Relational leadership and management, as opposed to hierarchical styles, can promote sustainability by encouraging harmony within and outside the practice [35].

Participants would often use the terms management and leadership together, describing the manager as the leader, but as displaying an inclusive leadership style. However, this leader-centric perspective may not be appropriate for all MGPs. The act of naming the leader because of the role they occupy may not be suitable for all managers or all situations. MGPs consist of a group of highly qualified, professional, passionate and enthusiastic midwives. It might be more appropriate to adopt a form of leadership that engages the individuals and the collective like leadership-as-practice [36]. Leadership-as-practice is less about what one person does and is more about what can be accomplished by a collective, through day-to-day experiences [37]. Managers that contribute to a collective leadership demonstrate that the midwives are valued, encouraging their involvement in decision-making, thereby improving job satisfaction [12].

According to the participants vigilance was essential. Vigilance helped to monitor outcomes, the direction of the MGP, and midwives’ wellbeing, without seeming to micromanage. Midwifing the midwives was a way of caring for the midwives in a similar vein to how good midwifery care supports women, underpinned by feminist values [31]. This study suggests that managers demonstrate their management as seemingly hands-off; yet the participants described the need for constant vigilance of the outcomes and midwives, again in a very similar way to good midwifery care. This vigilance allows managers to be aware of the outcomes and use these to support the model to protect and fight for the values of midwifery. These findings reflect those reported by others [38, 39] like Hewitt and colleagues [34] who found that managers were required to ‘hold the ground for women and midwives’, advocating for midwifery in a similar way that midwives advocate for women.

Midwifery care, which is often dominated by a biomedical approach and nursing models, is preoccupied with efficiency, promptness, and medically-regimented care [40]. A focus on time can disadvantage midwives and women [40, 41]. Models of care, like MGP, generally aim for: a slower approach to midwifery care; taking time to listen; and being with the woman. This allows the midwife to learn from the woman and build a relationship with her. Slow midwifery has been described as the midwifery angle on the slow food movement, valuing quality over quantity, a way of seeking balance in a fast-paced modern world. The participants described strategies that require the MGP manager to mirror slow midwifery. Strategies included taking time (not counting time), comforting, checking in on others, building capacity, and trusting [40].

MGP midwives trust that women will call if they need them; they trust in physiology, in women [42], and in each other [32]. The participants described how MGP managers also need to trust midwives’ judgement, work ethic, and expertise. Similarly, Hewitt and colleagues [34] found that managers needed to trust women and midwives, but also needed to be trustworthy. The participants in this study described the need for managers to trust that midwives will seek help when they need it and will discuss anything with them. Trust is consistent with the reciprocal relationship between the MGP midwife and manager, in a similar way that women and midwives trust in each other [42].

### The price of caring

Midwifery care requires being emotionally available to women and their families. While caseload midwives report higher satisfaction and less burnout than midwives providing fragmented care [19, 43], being on-call and a disrupted family life can cause stress and anxiety [44]. The participants recognised the need to be vigilant in supporting the midwives, helping them to manage stress and to be available to them when they needed to talk. This then requires the manager to also be emotionally available and to manage their emotions.

To help MGP midwives manage their stress, the participants described the need for MGP managers to be available, have an open-door policy, and to be welcoming. They also must not judge or show they disapprove, requiring the manager to keep outward appearances in check and to manage their emotions. MGP managers must also be attentive to the emotional state and stress levels of each individual midwife.

According to the participants, the ideal MGP manager carries a large emotional load, which can induce stress. Although managers’ emotional labour has not attracted as much research as frontline workers [45], managers’ emotional labour can produce stress and lead to job burnout [46]. Clarke and colleagues reported that managers’ emotional load is generally different to frontline workers; however, it is important that managers receive encouragement and acknowledgement of the emotional component of their work [46].

Emotional labour in midwifery, has been poorly recognised, unappreciated, and scarcely described [47]. Along with nursing, midwifery has historically been described as ‘women’s work’, and like many other female dominated roles, has been given little credit [48, 49]. While male work was seen as superior, important work, the caring work of midwifery and nursing was often viewed as easy [48]. This prompted a preoccupation with task-orientated care and the medical model taking carers away from relationship-based care and emotion [49].

Emotional labour can be described as the skill or art of caring, recognising the emotion in others and in self, while managing one’s own emotion [48]. In health care, feelings are often required to be induced or supressed to produce an acceptable outward appearance [49]. Emotional labour was coined by Hochschild [50] who recognised a discrepancy between the emotion demonstrated by air hostesses and the emotion they were feeling. The pretend empathy in the smile can take a toll on the individual, affecting how people listen to ‘feeling’ and their capacity to feel. Although the caring that midwives require in comparison to flight attendants may differ, emotional labour is nevertheless an invisible and rarely honoured source of stress [49]. However, providing empathy can provide the care giver with gratification known as compassion satisfaction [51].

The MGP manager is required to do a lot of juggling, involving communication, collaboration, and negotiation to keep the MGP afloat. Although the manager might display a calm exterior, this study found that the MGP manager constantly juggles – they juggle communication on many different levels, buffering the midwives from unsupportive stakeholders, educating, managing the budget, working with consumers, medicine, the hierarchy, and supporting the midwives. Hewitt and colleagues also reported juggling the forces [34]. The MGP manager’s many and varied requirements might also induce stress and require additional emotional labour. Because of the potential for increased stress, this study highlights the need for managers to be responsible for their own sustainable self-care.

### The role of self-care

Participants described the ideal MGP manager as someone who understands and deeply cares for the MGP. While it helps to have worked as a MGP midwife, it does not appear to be a necessity. What is necessary is that the MGP manager: is passionate about continuity of carer; deeply cares about the service and the midwives; and has feminist values with a strong woman-centred philosophy. Feminist values were displayed by striving for services that lead to equity and emancipation, like MGP for all women. The MGP manager must be able to juggle multiple forces within and outside the MGP [34], while giving the impression that they have the time to communicate with midwives, other disciplinary staff, their managers, managers of other departments, support staff, and consumers. Additionally, they need to be abreast of the MGP statistics, investigating and interrogating discrepancies or complaints, while keeping to budget and appeasing the dominant medical/nursing paradigms. This emotional and physical load requires support if the MGP management is to be sustainable. One of the participants described the need for the manager to take on duties that allow the midwives to deliver woman-centred care. In a similar way, MGP managers need support to enable them to provide midwife centred management.

However, many managers do not have the time to do their job properly due to conflicting priorities, where they are spread over several areas to manage. This has implications in the way they support midwives, and how they can manage stress. Managers are pivotal to the workplace culture and to the wellbeing of their staff, in turn reflecting on the care women and families receive [52]. The current crisis of the retention of midwives in the profession, calls for the managers’ workload to be taken seriously to enable them to support midwives to be healthy, happy and to stay in midwifery [10, 12, 52].

Self-care practices might not only save and sustain the manager; it might be something the manager can encourage within the MGP, which also helps to sustain the MGP. In a study on MGP midwives views regarding MGP management and sustainability, the midwives described the importance of self-care and of the manager supporting these practices [24]. While the health care facility might focus on budgetary requirements and accountability to the hierarchy rather than the consumers, the microcosm of the MGP could be a starting point for cultural change. MGP managers could encourage practices that promote taking care of, understanding, and healing the relationship an individual has with their bodies, with relevance to their personal and professional lives. Although one approach is unlikely to be universally appropriate, developing skills that encourage the individual to listen to subtle messages from their body, mind and spirit, through meditation practices might be a good place to start [53]. Another strategy for managers might be clinical supervision. Adcock and colleagues [39] found that clinical supervision, mentoring, and leadership education, can develop midwifery leaders and protect their emotional wellbeing.

### Strengths and limitations

A key strength of this study is the sample size where 23 midwives contributed, although participants only came from four Australian States and Territories. This number of participants represents considerable information power, given the in-depth qualitive nature of the study. Information power was described by Malterud and colleagues [54] as a way to determine sample size. A limitation of this study is that, due to the topic, many participants would have been attracted to participating because they are enthusiastic and passionate about the topic, giving a bias to the findings. This study helped to design a survey to provide more data on this topic and will hopefully encourage others to do more research in this area.

### Implications and future directions

Although this study was conducted to inform a large national survey, it has implications for researchers, policymakers, managers, midwives, and consumers. For researchers, this study highlights the need for more research on the management and leadership of progressive models of care, along with what can be done to ensure a more sustainable MGP workforce. It might also encourage more research into burnout and emotional labour in management. Policy makers, need to deliver policies that improve access to MGP and consider its sustainability given the improved outcomes for women and babies [7], the reduced burnout of midwives [55] and improved satisfaction for both women [18] and midwives [8, 12]. Service managers can support the implementation and sustainability of MGP by employing MGP managers that meet the criteria covered in this study and by giving them a sustainable workload. For midwives, this study reiterates the importance of self-care and support both at home and at work and how this can be developed within the culture of the MGP. For consumers, this study exposed the need for their input and feedback in driving and improving MGP services. Consumers should be involved from the start of MGP implementation through steering groups and working parties. They should be also involved with the ongoing direction of the service to ensure it continues to be a woman centred service.

## Conclusions

MGP managers are pivotal to sustain an MGP. The findings demonstrate the need for MGP managers to practice midwife-centred management and to have similar qualities to what is required for good midwifery care. This study also indicated that they experience emotional labour in their multifaceted position, which might benefit from the practice of self-care.

## Data Availability

The original transcripts from this study are not publicly available to ensure the individual privacy of the participants. Data are available on reasonable request from the corresponding author.

## Abbreviations

CMC: Clinical midwife consultant
MGP: Midwifery group practice
KPI: Key performance indicators
TA: Thematic analysis
